# A Triple Network Connectivity Study of Large-Scale Brain Systems in Cognitively Normal APOE4 Carriers

**DOI:** 10.3389/fnagi.2016.00231

**Published:** 2016-09-28

**Authors:** Xia Wu, Qing Li, Xinyu Yu, Kewei Chen, Adam S. Fleisher, Xiaojuan Guo, Jiacai Zhang, Eric M. Reiman, Li Yao, Rui Li

**Affiliations:** ^1^College of Information Science and Technology, Beijing Normal UniversityBeijing, China; ^2^State Key Laboratory of Cognitive Neuroscience and Learning, Beijing Normal UniversityBeijing, China; ^3^Banner Alzheimer’s Institute and Banner Good Samaritan PET CenterPhoenix, AZ, USA; ^4^Eli Lilly and CompanyIndianapolis, IN, USA; ^5^Center on Aging Psychology, Key Laboratory of Mental Health, Institute of Psychology, Chinese Academy of SciencesBeijing, China

**Keywords:** Alzheimer’s disease, APOE4, Bayesian network, connectivity, fMRI, triple network model

## Abstract

The triple network model, consisting of the central executive network (CEN), salience network (SN) and default mode network (DMN), has been recently employed to understand dysfunction in core networks across various disorders. Here we used the triple network model to investigate the large-scale brain networks in cognitively normal apolipoprotein e4 (APOE4) carriers who are at risk of Alzheimer’s disease (AD). To explore the functional connectivity for each of the three networks and the effective connectivity among them, we evaluated 17 cognitively normal individuals with a family history of AD and at least one copy of the APOE4 allele and compared the findings to those of 12 individuals who did not carry the APOE4 gene or have a family history of AD, using independent component analysis (ICA) and Bayesian network (BN) approach. Our findings indicated altered within-network connectivity that suggests future cognitive decline risk, and preserved between-network connectivity that may support their current preserved cognition in the cognitively normal APOE4 allele carriers. The study provides novel sights into our understanding of the risk factors for AD and their influence on the triple network model of major psychopathology.

## Introduction

The apolipoprotein e4 (APOE4) gene has been well established as a susceptibility gene for sporadic and late-onset familial Alzheimer’s disease (AD; Poirier et al., 1995; Reitz and Mayeux, 2010; Kandimalla et al., 2013; Tai et al., 2014). Epidemiologic evidence has clarified that APOE4 decreases the age-at-onset of AD in a gene dosage-dependent manner (Corder et al., 1993; Breitner et al., 1999). Neuroimaging studies have demonstrated that APOE4 carriers exhibit elevated medial temporal lobe (MTL) atrophy (Agosta et al., 2009; Fleisher et al., 2009a,b; Wolk and Dickerson, 2010), and recent studies have shown that the APOE4 allele is associated with Cerebrospinal fluid (CSF) biomarkers including Aβ42, tau (Kandimalla et al., 2011) and ubiquitin levels (Kandimalla et al., 2014). Thus the APOE4 allele has been suggested as an important factor that leads to lower cognitive performance, or the progression to mild cognitive impairment (MCI) and AD (Barabash et al., 2009; Sasaki et al., 2009).

Functional neuroimaging connectome studies of AD have proposed a disconnection hypothesis of the disease. Many studies have consistently reported that the cognitive impairment in AD and the cognitive decline in its preclinical stage were largely due to the disruptions of the brain networks (Stam et al., 2007; Lo et al., 2010; Wang et al., 2013). For example, as one of the most relevant networks in AD, various studies have shown that the default mode network (DMN) exhibited a disruption in functional connectivity in AD (Greicius et al., 2004; Rombouts et al., 2005; Celone et al., 2006; Petrella et al., 2007; Wu et al., 2011), and even at early stages of the disease such as MCI (Lustig et al., 2003; Rombouts et al., 2005; Celone et al., 2006; Petrella et al., 2007; Qi et al., 2010; Li et al., 2013). In addition to the DMN, other networks have also been found to show alterations in AD. For example, the salience network (SN), whose connectivity showed negative correlation with DMN has been linked to AD (Zhou et al., 2010; Balthazar et al., 2014). These alterations in functionally coordinated brain systems can occur long before disease onset in cognitively normal people with various risk factors for AD (Poirier et al., 1995; Kivipelto et al., 2001; Song et al., 2015). For example, Westlye et al. (2011) demonstrated a negative correlation between DMN synchronization and memory performance in healthy APOE4 carriers. Besides, the functional alterations in the DMN and SN connections were also demonstrated in the elderly APOE4 carriers (Machulda et al., 2011). These evidences suggested that the presence of APOE4 gene is accompanied by brain network alterations that are closely relevant to AD progression.

Recently, a triple network model of major psychopathology has been proposed by Menon (2011). The triple network model consists of the central executive network (CEN), SN and DMN. These three networks are generally referred to as the core neurocognitive networks due to their involvement in an extremely wide range of cognitive tasks (Greicius et al., 2003; Greicius and Menon, 2004; Menon and Uddin, 2010; Menon, 2011). Specifically, the CEN and SN typically show increased activation during stimulus-driven cognitive or affective processing, while the DMN shows decreased activation during tasks in which self-referential and stimulus-independent intellectual activity is not involved (Greicius et al., 2003; Greicius and Menon, 2004). The triple network model suggests that the aberrant internal organization within each functional network and the interconnectivity among them are characteristic of many psychiatric and neurological disorders. Recently the triple network model has been widely applied to elucidate the dysfunction across multiple disorders, including schizophrenia, depression and dementia (Menon and Uddin, 2010; Menon, 2011; Zheng et al., 2015; Yuan et al., 2016). However the triple network interactions in elderly APOE4 carriers who are at high risk to AD have not yet been explored.

In the present study, we investigated the APOE4-mediated modulation of the within-network functional connectivity and the between-network connectivity of the three core networks included in the triple network model in cognitively normal individuals carrying a family history of AD and at least one copy of the APOE4 allele using functional magnetic resonance imaging (fMRI). A group independent component analysis (ICA) approach and Bayesian network (BN) approach were used to separate the functional connectivity networks from the fMRI dataset and to determine the between-network effective connectivity, respectively.

## Materials and Methods

### Participants

fMRI data from 29 cognitively normal right-handed volunteers (8 males and 21 females, ages between 50 and 65 years) who were the subjects in our previous study (Fleisher et al., 2009b) were included in this work. They were divided into two groups: the high-risk group and the low-risk group. The high-risk group included 17 subjects who had a significant family history of dementia in a first-degree relative and at least one copy of the APOE4 allele. The other twelve participants who had neither a family history of dementia nor a copy of the APOE4 gene were regarded as the low-risk group. Notably, there were no significant differences in age, gender and education level between these two groups (all *ps* > 0.05). The two groups were matched on general cognitive function as evaluated by Folstein Mini Mental State Exam (*p* = 0.39). The study was conducted according to Good Clinical Practice, the Declaration of Helsinki and US 21 Code of Federal Regulations (CFR) Part 50-Protection of Human Subjects, and Part 56-Institutional Review Boards and was approved by the Institutional Review Board of the University of California, San Diego. Written informed consent for the study was obtained from all of the participants before protocol-specific procedures were performed, including cognitive testing.

All scans were performed on a General Electric Signa EXCITE 3.0 T short bore, twin speed scanner with a body transmit coil and an 8 channel receive array. High-resolution structural brain images were acquired with a magnetization prepared from three-dimensional fast spoiled gradient sequence acquisition (FSPGR: 124 axial slices, 1 mm×1 mm in-plane resolution, 1.3 mm slice thickness, Field of View (FOV) = 256 mm^2^ × 256 mm^2^, TR = 7.8 ms, TE = 3.1 ms, flip angle = 12°). Blood oxygen level dependent (BOLD) data were acquired using echo planar imaging sequences (35 slices, perpendicular to the axis of the hippocampus, 6 mm in-plane resolution, 0 spacing, FOV = 220 mm^2^ × 220 mm^2^, TE = 30 ms, TR = 2500 ms, voxel size = 3.4 mm^3^ × 3.4 mm^3^ × 6.0 mm^3^).

### Data Preprocessing

For each participant, the original first five-time functional images were discarded to allow for equilibration of the magnetic field. All of the preprocessing steps were performed using the Statistical Parametric Mapping program (SPM8^1^). They included within-subject inter-scan realignment, between-subject spatial normalization to a standard brain template in the Montreal Neurological Institute (MNI) coordinate space, and smoothing by a Gaussian filter with a full width at a half maximum of 8 mm. Following this, the linear trend with regard to time was removed by linear regression via the Resting-State fMRI Data Analysis Toolkit (REST^2^).

After the preprocessing, we employed the Group ICA and BN to learn the functional interactions of the triple network model. Group ICA was first used to isolate the three brain networks for examination of the functional connectivity changes within each network in the high risk group. The BN was then used to show the directed causal effects between these three networks in the high risk group. Thus, the study was developed to delineate the influence of APOE4 on the triple networks in both within-network connections and between-network interactions.

### Group Independent Component Analysis

Group ICA is widely used to separate patterns of task-activated neural networks, image noises, and physiologically generated independent components (ICs) in a data-driven manner. The preprocessed data of all participants were entered into the Group ICA program in the fMRI Toolbox (GIFT^3^) for the separation of the three networks included in the triple network model and the determination of networks for BN analysis. The Group ICA program included two rounds of principal component analyses (PCA) for reduction of fMRI data dimensions, ICA separation and back-reconstruction of the ICs (Calhoun et al., 2001). The optimal number of principal components, 31, was estimated based on the minimum description length (MDL). In the first round of PCA, the data for each individual subject were dimension-reduced to the optimal number temporally. After concatenation across subjects within groups, the dimensions were again reduced to the optimal numbers via the second round of PCA. Then, the data were separated by ICA using the Extended Infomax algorithm (Lee et al., 1999). After ICA separation, the mean ICs and the corresponding mean time courses over all of the subjects were used for the back-reconstruction of the ICs and time courses for each individual subject (Calhoun et al., 2001).

Finally, the ICs that best matched the CEN, DMN, and SN for both the low- and high-risk groups were selected separately. Following this, one-sample *t*-test (*p* < 0.001, corrected by family wise error (FWE)) was performed to determine the CEN, DMN, and SN functional connectivity for the low-risk and high-risk groups respectively. Between group within-network functional connectivity difference was determined by two-sample *t*-test (*p* < 0.05, corrected by false discovery rate (FDR)).

### Bayesian Network Analysis

BN analysis can be used to learn the global connectivity pattern for complex systems in a data-driven manner, and has been applied in our previous studies of AD and MCI (Wu et al., 2011; Li et al., 2013). Here, we employed the Gaussian BN method to characterize the large-scale networks in terms of directed effective connectivity among CEN, DMN and SN.

To establish the effective connectivity pattern of the three networks for the low- and high-risk groups separately, we defined the region of interest (ROI) mask as each of the three one-sample *t*-test network map (*p* < 0.001, FWE corrected). The averaged time series over these voxels in every subject was extracted and then entered into the BN analysis for the construction of an effective connectivity pattern of the three core networks.

A BN model is a directed acyclic graph that encodes a joint probability distribution over a set of random variables. The directed arcs in the graph denote the conditional dependence relationships between nodes, which are qualified by the conditional probability of each node given its parents in the network. Specific to our BN model, we have three nodes in total, which represent the three core networks in the triple network model, and the arcs connecting them represent the directed effective connectivity between these functional networks. The time series of each node was calculated as the mean time series in each network ROI, and was assumed to follow a linear Gaussian conditional distribution. To learn the effective connectivity of the triple network model, we employed the Bayesian information criterion (BIC)-based learning approach. The BN model that maximized the BIC score among the space of possible candidates was selected as the best fit network. We used the L1-Regularization Paths algorithm (Schmidt et al., 2007) and the Maximum Likelihood Estimation (MLE) implemented in the collections of Matlab functions written by Murphy et al.^4^ to learn the structure and parameters of the BN model, respectively, for the high- and low-risk groups.

### Effective Connectivity Comparison Between the High- and Low-Risk Groups

To examine the effective connectivity difference of CEN, DMN and SN between the high- and low-risk groups, we adopted the randomized permutation procedure. We used the differences of the connection weight coefficients between the two groups as the statistical measure. The reference distribution is obtained by calculating all possible values of the test statistic under rearrangements of the group labels on the observed fMRI datasets. The statistics for the real two group samples were calculated first. Then, at each iteration of the test process, the subject-group membership was randomly assigned for each subject. A BN model for each rearranged group was constructed, and the differences of the connection weight coefficients between the two rearranged groups were calculated. We ran a total of 1000 permutations and assessed the sample distributions for these statistics. Finally, for each of the connections presented in the BN model for the two risk groups, type I errors of having between-group differences were estimated.

## Results

### Functional Connectivity of CEN, DMN and SN

**Figure 1:**
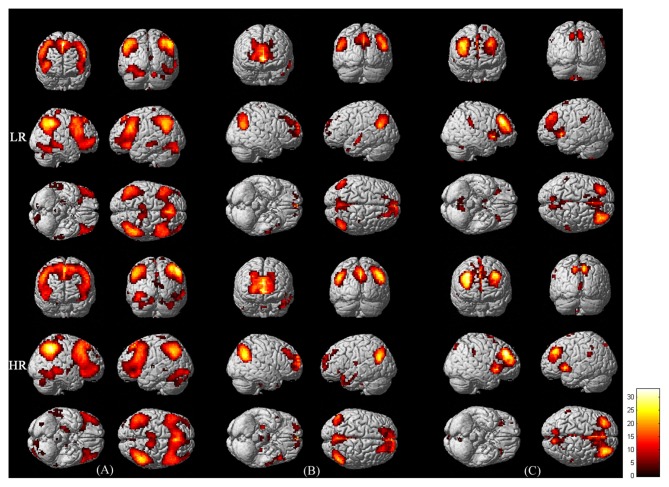
**Functional connectivity maps of the central executive network (CEN; A) default mode network (DMN; B) and salience network (SN; C) in LR (upper panel) and HR (lower panel) groups.** The maps were derived from the one-sample *t*-test of Group independent component analysis (ICA; *p* < 0.001, corrected by family wise error (FWE)). Bar at the right shows *T*-values.

Figure 1 shows the three networks included in the triple network model in the low and high-risk groups detected by Group ICA (one-sample *t*-test, *p* < 0.001, FWE corrected). In both groups, the CEN includes the dorsolateral prefrontal cortex and the lateral posterior parietal cortex. The DMN includes the posterior cingulate cortex, medial prefrontal cortex, bilateral inferior parietal cortex, inferior temporal cortex and the hippocampus. The SN includes the dorsal anterior cingulate cortex and the fronto-insular cortex.

### Within-Network Functional Connectivity Difference Between Groups

**Figure 2:**
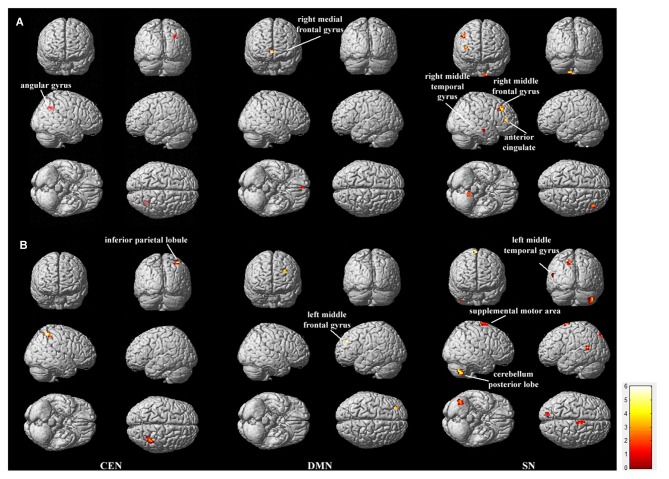
**Regions showing between-group functional connectivity difference.** The comparison was performed for each of the triple networks by the two-sample *t*-test with *p* < 0.05, false discovery rate (FDR) correction. **(A)** shows the regions in which functional connectivity are stronger in LR group than in HR group (LR > HR), and **(B)** shows the opposite case (HR > LR). Bar at the right shows *T*-values.

To compare the within-network functional connectivity difference of the CEN, DMN and SN between the low- and high-risk groups, we performed a two-sample *t*-test (*p* < 0.05, corrected by FDR) on individual maps of the three networks between the two groups. Figure 2 displays the functional connectivity differences between the low and high-risk groups.

Within the CEN, the angular gyrus displayed increased functional connectivity in the low-risk group compared with the high-risk group (“LR > HR”), whereas the inferior parietal lobule displayed increased functional connectivity in the high-risk group compared with the low-risk group (“HR > LR”). Within the DMN, the right medial frontal gyrus displayed increased functional connectivity in the low-risk group compared with the high-risk group (“LR > HR”), whereas the left middle frontal gyrus displayed increased functional connectivity in the high-risk group compared with the low-risk group (“HR > LR”). Within the SN, the regions including the right middle temporal gyrus, right middle frontal gyrus and the anterior cingulate cortex displayed increased functional connectivity in the low-risk group compared with the high-risk group (“LR > HR”). In contrast, the regions including the left middle temporal gyrus, posterior lobe of the cerebellum and the supplemental motor area displayed increased functional connectivity in the high-risk group compared with the low-risk group (“HR > LR”). Details on these regions with between-group functional connectivity differences are listed in Table 1.

**Table 1:** **Brain regions that showed functional connectivity differences between the low and high risk groups (two sample *t*-test, *p* < 0.05, corrected by false discovery rate (FDR))**.

Regions	L/R	*T* value	MNI coordinate			Number of voxels
			*x*	*y*	*z*	
Angular gyrus	R	5.40	30	−54	36	35
Middle frontal gyrus	R	5.90	48	33	44	51
Middle temporal gyrus	R	6.92	48	−15	−16	18
Anterior cingulate	R	5.74	9	30	20	62
Medial frontal gyrus	R	5.25	3	54	−4	15
Inferior parietal lobule	R	5.47	39	−48	60	69
Middle frontal gyrus	L	4.60	−30	45	28	29
Cerebellum posterior lobe	R	5.58	45	−63	−44	71
Middle temporal gyrus	L	5.17	−51	−51	16	21
Supplemental motor area	R	6.16	6	−6	76	42

### BN-Based Effective Connectivity of CEN, DMN and SN

**Figure 3:**
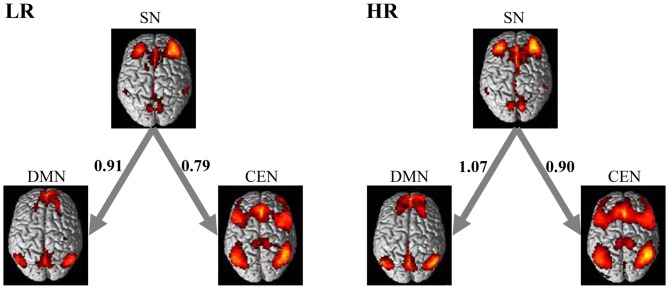
**Directed interactions of the triple networks in two groups.** The causal interactions were determined based on the Bayesian network (BN) analysis of the triple networks. The LR group and HR group were found to have the same triple network BN connectivity relationships. The SN plays as an influential hub that mediates the activity of the CEN and DMN in both groups. The numbers on the connections represent the BN connectivity weights between brain networks.

Figure 3 shows the effective connectivity of the CEN, DMN and SN in the low-risk group and high-risk group learned using Gaussian BN approach. In accordance with the triple network model (Menon, 2011), Figure 3 demonstrates consistently in the two groups that the DMN together with CEN receive connections from SN. It is important to note that the SN plays as a special node that does not receive but only generates connections in the model in both groups. Furthermore, the result of the random permutation test indicates that there is no significant difference among the effective connectivity coefficients of these three networks between the low- and high-risk groups (all *p*s > 0.05).

## Discussion

The focus of the present study was to explore the possible impairment of the within-network functional connectivity and the between-network effective connectivity of the large-scale triple networks in cognitively normal individuals with a family history of AD and at least one copy of the APOE4 allele. Group ICA of the triple network model found that a couple of brain regions in the three networks showed significantly altered functional connectivity in the high-risk individuals, while the BN analysis of the model did not find significant between-group difference in the causal connections among the three functional networks.

We first compared the within-network functional connectivity between the low-risk subjects and the high-risk subjects. The results demonstrated that a number of brain regions, including the medial prefrontal gyrus from the DMN, the angular gyrus from the CEN, the anterior cingulate, the right medial temporal and the right middle frontal gyri from the SN displayed significantly decreased functional connectivity in APOE4 carriers. The medial prefrontal gyrus is a critical area of the DMN (Greicius et al., 2003), and plays a central role in a variety of cognitive functions, especially memory (Euston et al., 2012) and executive function (Dalley et al., 2004) that are vulnerable to cognitive aging and AD (Greicius et al., 2004; Burke and Barnes, 2006; Li et al., 2013). Various studies of the DMN in AD have repeatedly reported functional connectivity disruption in this region (Greicius et al., 2004; Rombouts et al., 2005; Qi et al., 2010; Wu et al., 2011; Wang et al., 2013). Recently, Song et al. (2015) also demonstrated APOE effect on the medial prefrontal regions in the DMN using seed-based functional connectivity analysis. The angular gyrus is functionally related to associative memory (Ben-Zvi et al., 2015), visuo-spatial attention (Cattaneo et al., 2009), and language ability (Bernal et al., 2015). Agosta et al. (2012) have reported decreased functional connectivity of the angular gyrus from the fronto-parietal CEN in AD. Disrupted functional connectivity of the SN was associated with cognitive and emotional deficits, and has been found in advanced aging and MCI patients (He et al., 2014; Uddin, 2015; Lu et al., 2016). Recently, Joo et al. (2016) and Wang et al. (2015) investigated the functional disruptions in these functional networks, and found that greater reductions of inter-network connectivity were associated with lower cognitive performance in different levels of cognitive impairment. Thus the result here indicated that the functional connectivity in the triple networks was different between the high- and low-risk groups, which may be related to the presence of APOE4 and a family history of dementia. We speculate that these AD-like functional connectivity disruptions in the triple network model may suggest risks of future cognitive decline or the progression to MCI or AD for the APOE4 carriers.

In contrast with the decreased functional activation compared with the low-risk group, we found that the high-risk group also showed increased functional activation in the frontal gyrus, parietal lobe, temporal gyrus and the cerebellum. It is consistent with several recent neuroimaging studies of APOE effects on brain connectivity. For example, Machulda et al. (2011) found increased SN connectivity by calculating the functional connectivity of the anterior cingulate seed in APOE4 carriers. Westlye et al. (2011) and Song et al. (2015) demonstrated increased DMN synchronization in APOE4 carriers. Similarly in AD patients, increased functional activation compared with that in healthy controls has also been reported (Wang et al., 2007; Qi et al., 2010; Zhou et al., 2010; Li et al., 2013). These increases have been usually interpreted as a compensatory reallocation or recruitment of brain resources (Cabeza et al., 2002), which may be a protective factor to keep retain a normal cognitive level in individuals at high risk for AD.

We also employed a BN approach to model and compare the effective connectivity patterns between the CEN, SN and DMN in the low- and high-risk groups. The BN learning approach revealed same-directed connections and network features in these two groups; the SN node does not receive but only generates connections to CEN and DMN. The BN-based directed connectivity pattern in both groups is consistent with the triple network model of major psychopathology suggested by Menon (2011), in which the information transfer occurs only from the SN to the CEN and DMN. It is also consistent with the study of Uddin et al. (2011), in which they employed Granger causality analyses to model the effective connectivity of the triple network with development, and found consistently that the fronto-insular cortex in the SN significantly influence the functional activity of regions in the DMN and CEN. Moreover, a recent study of Liang et al. (2015) demonstrated that the topological organization of the triple network changes with cognitive task loads. By comparing the effective connectivity coefficients between these two risk groups via the random permutation test, however, we found no significant difference in the directed connectivity of the three networks between the low- and high-risk groups. It suggested that although the APOE4 carriers might demonstrate AD-like functional connectivity changes in each of the three networks, the interactions between them could retain a normal process as in non-APOE4 carriers. This interesting finding may be explained first by the methodological difference. The functional connectivity stresses the temporal correlation between different regions, while the effective connectivity refers explicitly to the causal influence that one system exerts over another (Friston, 2011), which is in accordance with the inherent meaning of the triple network model. Second, the BN-based directed connectivity reflects how these three networks in the model cooperate with each other to execute tasks. It essentially demonstrated an organizational architecture of these functional networks. We propose that the stable effective connectivity architecture of the triple networks may be a crucial factor, together with the increased within-network functional connectivity, that enables individuals at high risk for AD to retain a normal cognitive level. Finally, it might be related to the complexity of brain network itself in response to the APOE4 effect. We speculate that the within-network regional connectivity alterations might emerge earlier than between-network changes, and the further deterioration of within-network connectivity may gradually lead to disruptions in interactions between networks for the APOE4 carriers. For example, Zhu et al. (2016) recently reported more changes of within-network connectivity than between-network connectivity in AD and MCI. Further studies would be required to investigate the dynamic changes of the directed connectivity architecture of the triple networks in APOE4 carriers through a longitudinal study.

In summary, we have explored the functional connectivity and effective connectivity of the three networks included in the large-scale triple network model in individuals with low and high risk for AD. The results demonstrated aberrant within-network functional connectivity that suggests future risk of cognitive decline or progression to AD, and preserved between-network effective connectivity that may support their current preserved cognition in the cognitively normal individuals who have a family history of AD and at least one copy of the APOE4 allele.

## Author Contributions

XW, LY and RL: designed and wrote the article; KC, ASF and EMR: carried out the experiment and collected the data; QL and XY: analyzed the data; XG and JZ: participated in the discussion and criticized the manuscript.

## Conflict of Interest Statement

The authors declare that the research was conducted in the absence of any commercial or financial relationships that could be construed as a potential conflict of interest.

## Footnotes

^1^http://www.fil.ion.ucl.ac.uk/spm

^2^http://restfmri.net

^3^http://icatb.sourceforge.net/

^4^https://code.google.com/p/bnt
